# An Approach to Model Based Testing of Multiagent Systems

**DOI:** 10.1155/2015/925206

**Published:** 2015-03-22

**Authors:** Shafiq Ur Rehman, Aamer Nadeem

**Affiliations:** Center for Software Dependability, Mohammad Ali Jinnah University, Islamabad 44000, Pakistan

## Abstract

Autonomous agents perform on behalf of the user to achieve defined goals or objectives. They are situated in dynamic environment and are able to operate autonomously to achieve their goals. In a multiagent system, agents cooperate with each other to achieve a common goal. Testing of multiagent systems is a challenging task due to the autonomous and proactive behavior of agents. However, testing is required to build confidence into the working of a multiagent system. Prometheus methodology is a commonly used approach to design multiagents systems. Systematic and thorough testing of each interaction is necessary. This paper proposes a novel approach to testing of multiagent systems based on Prometheus design artifacts. In the proposed approach, different interactions between the agent and actors are considered to test the multiagent system. These interactions include percepts and actions along with messages between the agents which can be modeled in a protocol diagram. The protocol diagram is converted into a protocol graph, on which different coverage criteria are applied to generate test paths that cover interactions between the agents. A prototype tool has been developed to generate test paths from protocol graph according to the specified coverage criterion.

## 1. Introduction

Autonomous agents possess features like reactivity and proactivity, and they are able to interact with each other in order to perform certain tasks. Multiagents systems are used in complex application due to agent's unique features. Agents perceive their environment and respond accordingly to meet their goal. Autonomy is the agent's ability to operate independently, without the need for human guidance or intervention [1]. Application of multiagent systems is seen in many domains like e-commerce, banking, air traffic control, information management, and so forth. There are many agent development methodologies in which agent based systems can be modeled; one of them is Prometheus methodology [2]. Prometheus agent oriented software engineering methodology has a well-developed process from system specification to architectural design and then detailed design leading easily to code.

The term autonomy refers to the goal oriented behavior of agents. Autonomous agents are programmed to perform automatically in order to achieve certain goals. All of their activities converge towards achieving their defined goals. There are certain commercial agent applications presented in [3] which show the sensitivity of agent applications as they are meant to solve the real life problems in almost every domain. Real-time response and dynamism make testing of such application very hard. Performance and accuracy of results must be checked and this can be achieved with the effective testing of agent applications.

Padgham and Winikoff show that agent systems provide great flexibility, with over a million ways to achieve a given goal using only a relatively small hierarchy of goals and plans [4]. Because agents are autonomous and flexible, agent systems can be difficult to test. Therefore an approach is necessary that can test an agent system effectively and efficiently.

Prometheus is a methodology for designing intelligent agents from specification to detailed design and implementation [2]. One can model the agent using the Prometheus methodology starting from system specification to detailed design which includes identifying environment or external actors and scenarios with details of actions and percepts involved. Scenarios have actions and percepts associated with them. Different agents are responsible for different goals and different plans are associated with different goals [5]. Prometheus also supports the design via tool named Prometheus Design Tool (PDT) [6] in which design activities can be modeled. We can capture the relationship between goals and plans of an agent by goal-plan diagram. We have demonstrated this relationship in our paper [7]. Interaction protocol in detailed design is captured by interaction pattern/sequence between the agents in a certain scenario. These interactions occurred between agents and actors in form of messages, actions, and percepts. Agent systems to perform correctly these interactions must be tested and their occurrence in protocol must be verified with test data. Based on autonomous agents testing we have two research questions which we will cover in our proposed testing framework.

(i) How can design artifacts be used to test the interactions in a multiagent system?

This involves illustrating how design artifacts are chosen to be used to test the different agent interactions. Each interaction is carried out to meet some defined goals. We can extract goal-plan diagram as we discussed in our earlier research [7] and use the flow between goals and plans with respect to agent interaction. Interactions between agents and actor include message, action, and percept. Only message interactions are covered in [8]; actions and percepts have not been covered. We use the protocol diagram and convert it to protocol graph to test all sort of interactions between the agents and actor in specific protocol.

(ii) How can the process of generating such tests be guided by coverage criteria?

Define the scope coverage of the testing framework; identify additional coverage criteria with existing criteria discussed in [8] and probably identifying the additional coverage criteria which will cover action and percepts as well in any interactions diagram.

Our aim is to test the interactions of agents using their model specified in terms of interaction protocol. We have developed a tool which generates test paths based on specified coverage criterion that will test the interaction between the agents via some protocol. In order to achieve a goal there can be interactions between the agent and environment as well.

An agent achieves its goal with the help of plans specified. A main goal may have some subgoals contributing their part in achieving the objective. A goal-plan diagram can be used to describe the behavior of the agent showing all relevant actions, percepts, messages, and subgoals to be performed during the execution.  Section 2 describes modeling methodologies and how Prometheus is a better approach to design multiagent system.  Section 3 focuses on related work done in testing of autonomous agents.  Section 4 describes the details of testing framework for model based testing of autonomous agents. In Section 5 a case study has been presented by applying our testing framework.  Section 6 describes the conclusion and future work; references are shown at the end.

## 2. Modelling Methodologies

There are several agent-oriented software engineering methodologies, for example, Gaia (Generic Architecture for Information Availability) [9], Multi-Agent Systems Engineering (MaSE) [10, 11], MESSAGE [12], Prometheus [2], Tropos [13], CoMoMAS [14], SODA (Societies in Open and Distributed Agent spaces) [15], DESIRE [16], MAS-CommonKADS [17], and Belief-Desire-Intention (BDI) Model [18]. A methodology is collection of activities used to develop the system. Additionally methodology can be supported by the tool as well.

Agent architecture shows the behavior of agents, one of which is Belief-Desire-Intension (BDI) architecture [19]. BDI agents have certain goals to achieve. Belief-Desire-Intention properties are used to program intelligent agents. BDI agents have been widely used since last two decades and various researchers have explored their behavior. The agents whom we will discuss and use in our research are BDI agents. We consider multiagent systems developed by using Prometheus methodology. Padgham and Winikoff present Prometheus as an agent oriented methodology based on BDI agents [2].

Requirements are assumed to be known in Gaia methodology which forms the basis of analysis and design phases. Gaia is a methodology which distinguishes between analysis and design phases. It has Role Model and Interaction Model in analysis phase and Agent Model, Services Model, and Acquaintance Model in Design phase. Gaia has no tool support [9]. MaSE is an extension of the object-oriented approach that has two phases of analysis and design. MaSE does not have the view that agents should be autonomous and instead it assumes agents as only software which interacts with other softwares, that is, agents. Analysis contains three steps, that is, Capturing Goals, Applying Use Cases, and Refining Roles and design contains four steps, that is, Creating Agent Classes, Constructing Conversations, Assembling Agent Classes, and System Design [10]. MESSAGE adopts the life-cycle model of the Rational Unified Process (RUP) and is limited to analysis and design activities only. It uses UML as modeling language. It has five different views, for example, Organization view, Goal/Task view, Agent/Role view, Interaction view, and Domain view [12].

The Prometheus methodology [2] is a detailed AOSE methodology, which aims to cover all of the major activities required in the developing agent systems from system specification to architectural design and detailed design as well. Tropos is an AOSE methodology whose main distinction is the early requirement analysis. Agent related concepts like goals, plans, and tasks are included in all phases. No detailed information is available for last process defining agent types and mapping them to capabilities. The methodology does not appear to provide heuristics for any phase [18].

CoMoMAS focus in knowledge engineering problem arises in multiagent systems and provides extension in Cooperation Modeling Language for agents [14]. SODA focus on social inter-agent aspects of agent systems and that employs the concept of coordination models [15, 20]. DESIR contains expertise model and agents. Once analysis phase has been done, DESIRE could be used for specifying the design and implementation [16].

## 3. Related Work

To gain confidence on a multiagent system, it must be properly tested. Testing of software agent is an important and critical task as agents possess dynamic behavior. Basic agent-oriented concepts, for example, autonomy, mental attitudes, pro-activeness, and so forth, have been covered in the above discussed methodologies but there are several exceptions. Tropos was not perceived as being easy to use whilst MESSAGE and GAIA were both ranked weakly on adequacy and expressiveness. MaSE does not provide detailed design. Prometheus methodology is rich enough to provide detailed design and tool support as well for developers [18]. There is a need of quality assurance issues to be addressed in multiagent systems designed in Prometheus methodology. We are aiming to fill the gap of providing quality assurance and testing support for the multiagent systems designed using Prometheus methodology.

Agents have run time response and adaptability. Coverage criteria for testing can be applied to both code and model [21]. Code base conform that all code are covered in term of statements, and so forth while model based coverage requires the different interaction from different states of the system, represented in specific model [22].

Low et al. consider test coverage criteria for BDI agents [23]. They derive two types of control-flow graphs: one with nodes, where node represents plans for BDI agent and arcs present messages or other events which initiate certain plan, and another CFG in which node presents statements within plans and arcs represent control-flow between statements (a standard control-flow graph). Several coverage criteria are defined, based on node, arc, and path coverage and some were based on the success or failure of executing statements and plans [23]. Different interactions between the modeling artifacts are not presented. Instead this approach is not considering interactions between agents; our approach considers agent interactions in multiagent systems.

Zhang et al. presented an approach for model based testing for agent system [24]. Testing framework caters the different sequence of agent program execution. Fault directed testing approach is used by first identifying appropriate units of the agent and testing the unit with the defined mechanism. It considers the plan as a single unit; then it is checked whether the plan is triggered by the appropriate event or not, and its precondition, cycles in plan, and plan completeness, and so forth are checked. Event testing is performed for numbers of applicable plans for the event. An electronic bookstore system has been used as the sample system; testing framework will execute test units in a sequence [24]. No coverage measures have been taken while considering interactions between agent and external agent or stub. We are considering interactions between multiagent systems through coverage measures.

Zheng and Alagar proposed a method for conformance testing of agent's BDI properties as alternative to formal verification [25]. Test cases are generated to check the implementation with respect to specification. Winikoff and Cranefield have analyzed the size of behavior space for BDI agent and found that failure handling has larger impact on size of behavior space than expected [1]. Failure handling has been introduced in context of agent's behavioral space [1]. Both techniques above do not consider interactions between agents neither have any coverage measures been taken even in unit testing.

Nguyen et al. build an approach in which autonomous agents are tested with the help of evolutionary algorithm techniques in which test cases are represented as chromosomes [26]. Soft goals are used as the evaluation criteria so that test cases will be developed keeping in mind to meet the identified soft goals criteria to test the agent [26]. Each test case is evaluated through a defined fitness function. Goals are represented by quality functions and new tests are selected by reproduction. A framework for testing of autonomous agents has been presented in [27]. Individual agents have been tested in [26] and genetic algorithm idea on testing has been presented. Above technique does not cover multi-agent systems neither interactions between agents. We will test multiagent system and cover interactions between them; we are inspired to use genetic algorithm in our future extension.

Miller et al. state that the interaction between the agents possesses complex behavior and therefore testing of interactions is important [8]. They defined two sets of test coverage criteria for multiagent interaction testing. The first uses only the protocol specification, while the second considers also the plans that generate and receive the messages in the protocol [8].

Existing model based testing techniques for multiagent systems do not cover every aspect of multi-gent systems, that is, dependencies and interactions. Interactions between agents in Prometheus methodology have action and percepts interactions between agents as well which have not been covered still in existing techniques. Our approach to multiagent system testing covers such interactions as well and testing coverage will be done.

## 4. Proposed Testing Framework

In this section, we discuss our proposed approach for testing of multiagent system using the Prometheus design artifact defined in Prometheus Design Tool (PDT). Our proposed testing framework will address the automated test case generation of multiagent system using design artifacts. Interaction protocols will be used to build a test model which covers messages, actions, and percepts in order to achieve certain goal. Coverage criteria have been defined on protocol graph, covering every possible interaction between agents. In future we will test generated test paths with test data. Test data generation will be done with evolutionary algorithms. An algorithm for automated test case generation will be proposed and tool has been developed which uses identified coverage criteria, keeping in mind the messages and percepts and interaction protocol, and generates test paths.

**Figure 1:**
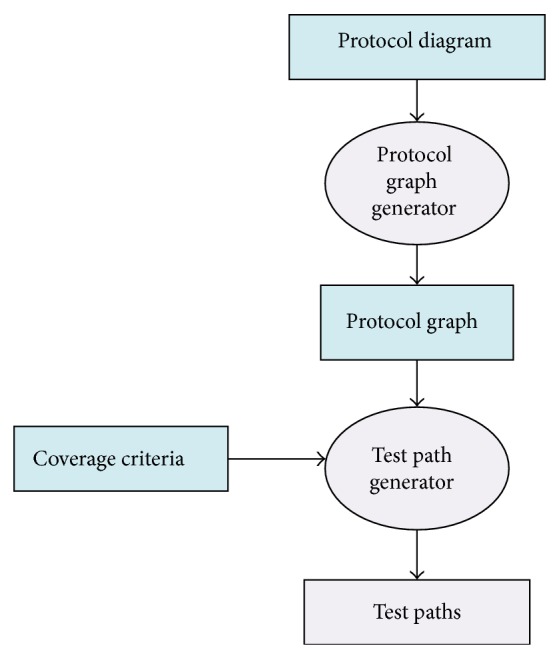
Proposed technique architecture.

**Figure 2:**
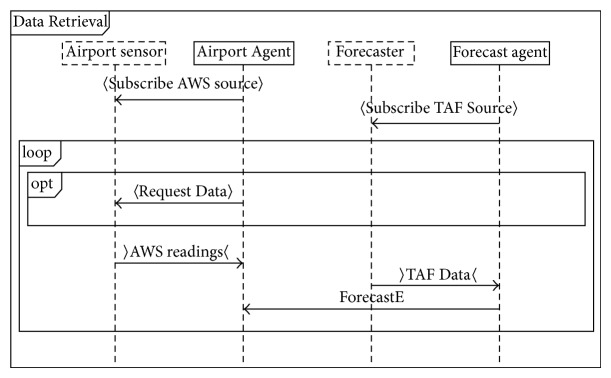
Data retrieval protocol diagram.

**Figure 3:**
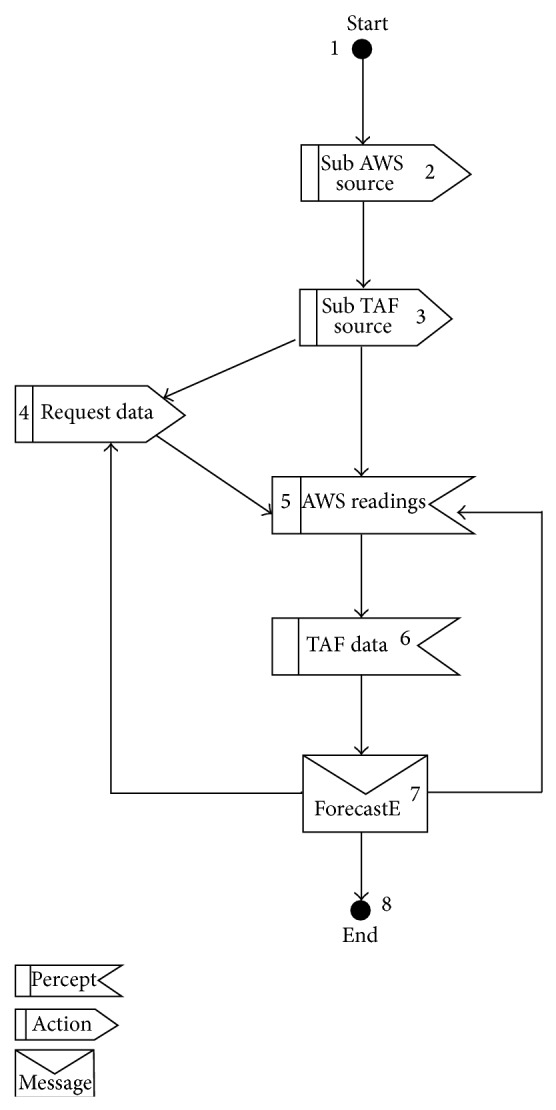
Protocol graph for data retrieval protocol diagram.

Figure 1 describes the testing framework of proposed technique. Our proposed technique has two main processes namely Protocol Graph Generator (Design Model) and test path generator. Design Model Generator uses Prometheus interaction protocol presented in protocol diagram (Figure 2) and generates a protocol graph (Figure 3) from it. The generated protocol graph gives a complete representation of all messages; percepts and action perform between the agents and actors in a specific protocol. Different coverage criteria will be defined focusing on percepts and actions as well along with messages and used as input to test path generator. Coverage criteria have been defined covering all possible interactions occurring in protocol graph. Test path generator uses protocol graph and applies different defined coverage criteria to generate test paths. Test paths will be generated using our test model, that is, protocol graph which will cater for interactions, messages, actions, and percepts in order to achieve certain goal.

Currently only message coverage criteria have been proposed by [8]. In a certain protocol, percepts and actions in an interaction have their importance and their coverage is necessary for effective testing. Our approach will uncover the interaction faults that would lie between the agents and actors.

### 4.1. Protocol Graph Generator

In our proposed testing framework, interaction protocol or protocol diagram is used as the design artifact which is transformed into a protocol graph. Protocol diagram contains messages, actions, and percepts interactions between agents and actors. Messages are passed only between the agents while actions and percepts interactions are performed between agents and actors.

#### 4.1.1. Protocol Diagram to Protocol Graph

Protocol diagram shows details of how messages, action, and percepts are involved in a protocol. In our work we convert the protocol diagram into protocol graph. Protocol graph has been introduced by Miller et al. [8]. They defined two sets of test coverage criteria for multiagent interaction testing. The first uses only the protocol specification, while the second considers also the plans that generate and receive the messages in the protocol. Miller et al. [8] do not cover the actions and percepts during the interaction. We have extended the protocol graph with actions and percepts as they are a very important part of interaction protocol.  Algorithm 1 is used to convert protocol diagram into protocol graph. We take protocol diagram as input and protocol graph has been produced by following Algorithm 1. Protocol diagram is represented in AUML representation as well.  Code 2 shows AUML description of protocol graph. Protocol graph represents interaction protocol in nodes and vertices form, on which different coverage criteria have been applied.

Once we have successfully converted protocol diagram into protocol graph, we need to generate test paths from protocol graph.  Algorithm 2 is used to generate test path from protocol graph.

### 4.2. Test Paths Generator

In this subsection we describe second process of our proposed approach named test path generator. Test path generator takes protocol diagram and coverage criteria as input and generates test paths for protocol. We have designed a test path generation Tool for automated test path generation. Coverage criteria have been defined in the following section.

#### 4.2.1. Test Coverage Criteria

Our aim in this research paper is to test the interaction done in a protocol; those interactions can be in form of message, action, or percept. Miller et al. [8] have proposed some coverage criteria on protocol graph like massage coverage and pair wise message coverage which are more likely the same.

**Figure 4:**
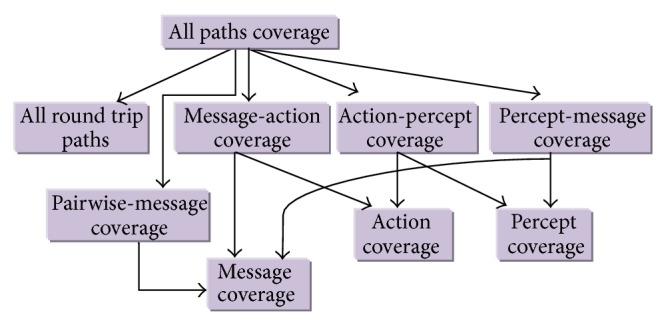
Test Coverage Criteria Hierarchy.

Additional coverage criteria for protocol graph including actions and percepts have been defined in testing technique. We have defined the following coverage criteria that will cover all possible aspects of interactions between agents and actors in the form of message, action, and percept.  Figure 4 shows hierarchy of test coverage criteria used to test multiagent system.

*Test Path*. A test path is a complete path in a protocol graph *G* that starts at node *i* and ends at node *f*. In following definitions of coverage criteria, *M* represents the set of all messages, *P* represents set of percepts and *A* represents set of all actions.

*(1) Message Coverage*. A set of test paths (TP) is said to satisfy message coverage criterion for a protocol graph *G* if each message node *m* of graph *G* is included in at least one path *P* ∈ TP.

This coverage criterion ensures that every message in protocol has been traversed at least once. There exists path from start to traversing all messages in it. 

*(2) Action Coverage*. A set of test paths (TP) is said to satisfy action coverage criterion for a protocol graph *G* if each action node “*a*” of graph *G* is included in at least one path *P* ∈ TP.

In this coverage criteria every action included in protocol graph must be included in generated test path for action coverage criterion. 

*(3) Percept Coverage*. A set of test paths (TP) is said to satisfy percept coverage criterion for a protocol graph *G* if each percept node *p* of graph *G* is included in at least one path *P* ∈ *TP*.

In this coverage criteria every percept included in protocol graph must be included in generated test path for percept coverage criterion. 

*(4) Message-Action Coverage*. A set of test paths (TP) is said to satisfy message-action coverage for protocol graph *G* if for each edge (*m*, *a*) in *G*, there is a test path *P* ∈ TP that contains subpath (*m*, *a*), where *m* ∈ *M* and *a* ∈ *A*.

Messages are passed between the agents and actions are passed between the agent and actor. Agent sends a message to an agent and agents send the action to actor; this sort of interaction must also be covered assuring the message-action coverage criterion.

*(5) Action Percept Coverage*. A set of test paths (TP) is said to satisfy action-percept coverage for protocol graph *G* if for each edge (*a*, *p*) in *G*, there is a test path *P* ∈ TP that contains subpath (*a*, *p*), where *a* ∈ *A* and percept *p* ∈ *P*.

Agents send an action to an actor in multiagent system demanding some task to be completed; in return actor sends the percept containing the required information or data, and this sort of communication is covered in action percept coverage criterion. 

*(6) Percept-Message Coverage*. A set of test paths (TP) is said to satisfy percept-message coverage for protocol graph *G* if for each edge (*p*, *m*) in *G*, there is a test path *P* ∈ TP that contains subpath (*p*, *m*), where *p* ∈ *P* and *m* ∈ *M*.

While receiving the percept from the actor, agents send a message to agent with necessary information; this sort of communication is covered in percept-message coverage criterion.

*(7) Pairwise-Message Coverage*. A set of test paths (TP) is said to satisfy pairwise-message coverage for protocol graph *G* if for each edge (*m*, *n*) in *G*, there is a test path *P* ∈ TP that contains subpath (*m*, *n*), where *m* ∈ *M* and *n* ∈ *M*.

In protocol graph, all cases in one message can be followed by another message are covered in pairwise-message coverage. Addition of pairwise-message coverage assures arc coverage which is left in message coverage criterion. 

*(8) All Round Trip Paths*. A set of test paths (TP) is said to satisfy all round trip paths coverage criterion for a protocol graph *G* if it loops back on same state in graph *G* in at least one test path *P* ∈ TP.

Interaction protocol describes the protocol in AUML protocol diagram which contains loops as well depending upon the protocol requirements. All round trip paths coverage criterion in protocol diagram traverse all loop at least once and include those paths which loops back on same state in generated test paths. 

*(9) All Paths Coverage*. A set of test paths (TP) is said to satisfy all paths coverage criterion for protocol graph *G* if it traverses every complete path *P* ∈ TP in *G* at least once.

All paths from start to end in a protocol graph are covered in all paths coverage criterion.

## 5. Case Study

**Figure 5:**
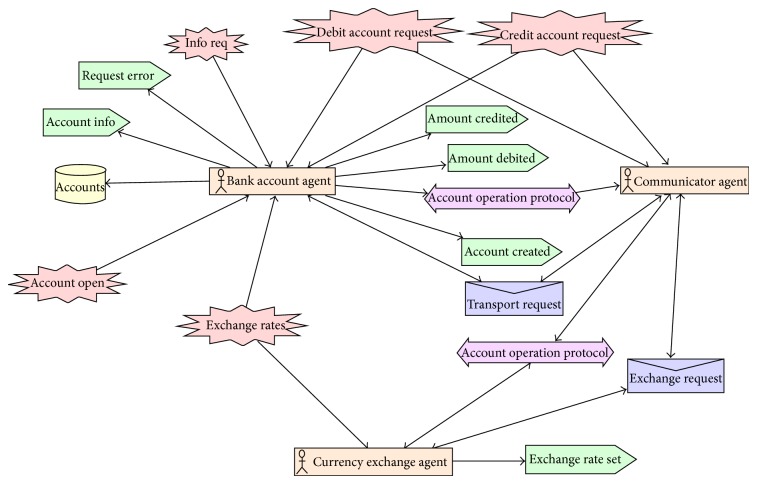
System overview diagram of multiagent system.

In this research paper we have taken case study of multicurrency Bank Account system [28] which maintains bank accounts in nominated currencies and performs currency conversions to allow transactions against the accounts to occur in any currency. It consists of a BankAccount agent, a CurrencyExchange agent, and a Communicator agent which acts as an interface [28]. We have designed the system overview diagram of account case study using Prometheus Design Tool [6].  Figure 5 shows system overview diagram of multiagent system in which different agents have interacted with each other via account operation protocol. Each agent has actions, percepts, and messages associated with it. Different interactions between agents and actors are occurring through account operation protocol as depicted in Figure 5.

Each protocol includes different interactions between agents and actors to perform specific tasks; such interactions are modeled in protocol diagram. Content of protocol diagram includes alternatives and loops and other deviations from a simple sequence are depicted in AUML using nested boxes [29].  Code 2 shows AUML description of account operation protocol diagram used in Prometheus Design Tool.

**Figure 6:**
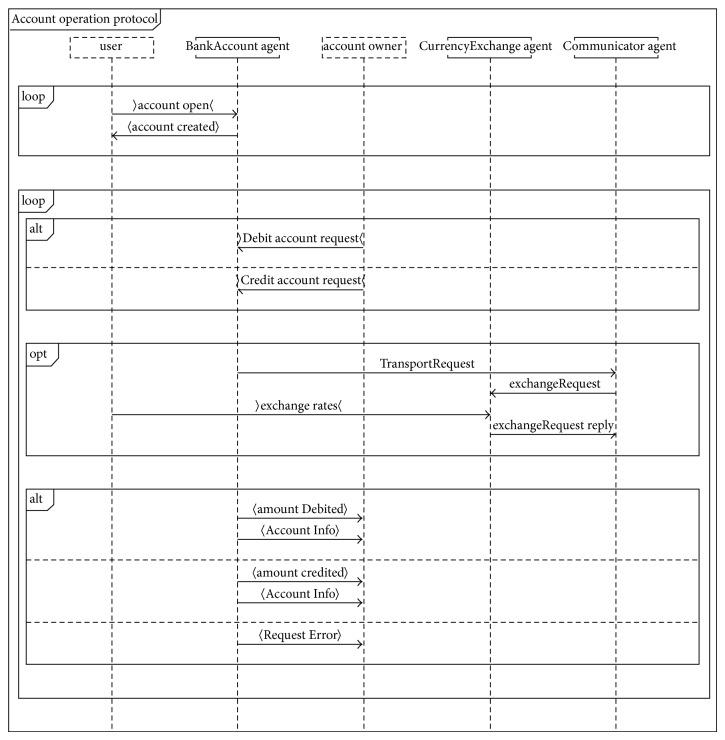
Account operation protocol diagram.

**Figure 7:**
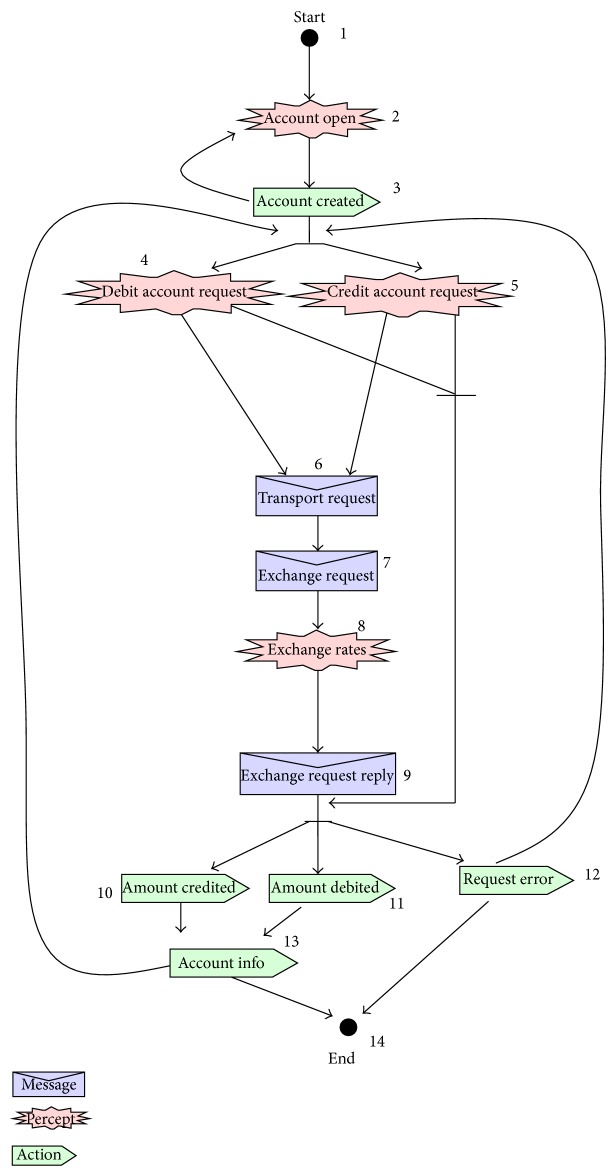
Protocol graph for account operation protocol diagram.

Figure 6 shows details of account operation protocol diagram [30] that is further converted to protocol graph (Figure 7) by protocol graph convertor process.

Table 1 shows test path against each coverage criteria we have defined and applied on our case study.

### 5.1. Test Path generator Tool

**Figure 8:**
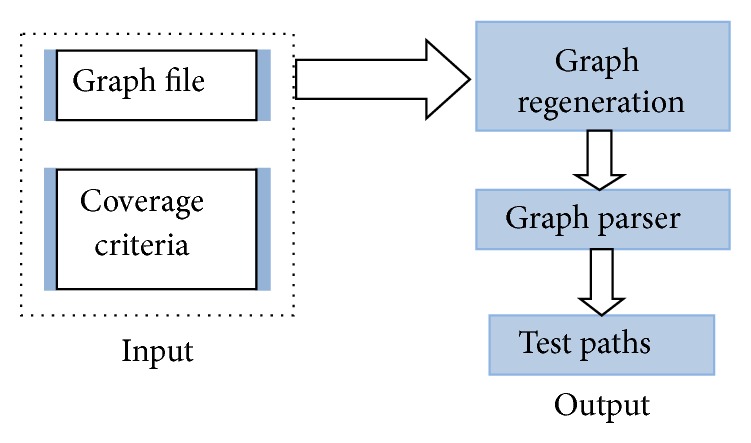
Test path generator tool process.

We have developed a tool to illustrate our proposed approach. Protocol diagram converted to protocol graph on which different coverage criteria have been applied to generate paths with respect to coverage criteria defined above. Our tool takes protocol diagram as input and generates test paths. Test path generator tool has two main classes namely Graph Regeneration and Graph Parser. Graph Regeneration reads the input file and makes a graph object according to the file. This object is used in the program to produce the paths. Graph Parser searches all the possible paths according to the coverage criteria given to it.  Table 2 shows input file for test path generator tool and Figure 8 shows the process of test path generation tool.

Different coverage criteria precondition and outcomes are programmed with respect to protocol graph.  Code 1 shows function which calculates all paths from protocol graph and coverage criteria are used to extract relevant path from all paths.

**Code 1: Find_all_paths function.**
**def** find_all_paths (names, graph, start, end, pathof=
      [“start”, “end”, “message”, “action”, “precept”], path=[]):
  path = path + [start]
  
**if** start = = end:
  
**return**  [path]
  
**if not** graph.has_key (start):
  
**return**  []
  paths = []
  
**for** node **in** graph [start]:
  
**if** names [node][1]**in** pathof:
   
**if** path. count(node) <2:
     newpaths = find_all_paths (names, graph, node, end, pathof, path)
     
**for** newpath **in** newpaths:
      paths. append (newpath)
  
**return** paths

**Code 2: AUML description of account operation protocol.**
start account operation protocol
actor A user
agent B BankAccount agent
actor C account owner
agent D CurrencyExchange agent
agent E Communicator agent
   box loop
   percept A B account open
   action B A account created
   end loop
   box loop
 box alternative
   percept C B Debit account request
  next
   percept C B Credit account request
   end alternative
   box opt
   message B E TransportRequest
   message E D exchangeRequest
   percept A D exchange rates
   message D E exchangeRequest reply
   end opt
   box alternative
   action B C amount Debited
   action B C Account Info
   next
   action B C amount credited
   action B C Account Info
   next
   action B C Request Error
   end alternative
   end loop
finish

**Figure 9:**
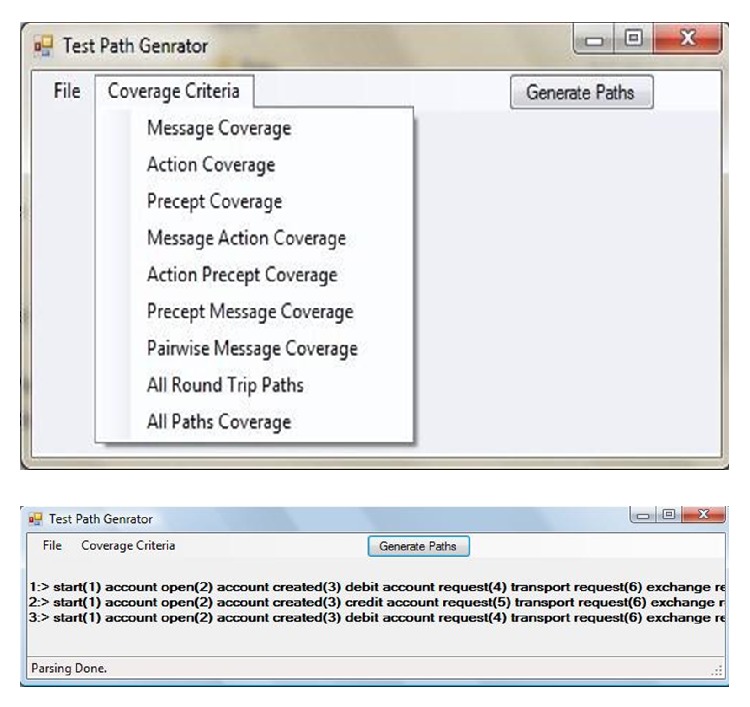
Test path generator tool.

Protocol graph contains the sequence of percepts, action, and message as described in corresponding protocol diagram of a certain interaction protocol.  Figure 9 shows the screen shot of our tool which automates the test path generation from design artifact like protocol graph.

## 6. Conclusion and Future Directions

In this paper, we have proposed a novel approach to test multiagent systems based on design artifacts following Prometheus methodology. Testing a multiagent system is a challenging task due to dynamic behavior of agents. Agents interact with each other and actors via some protocol. Interaction protocol diagram contains all sorts of interactions between agents and actor like message, action, and percepts. We have proposed a testing framework which transforms the interaction protocol diagram to a test model named protocol graph. The previously proposed protocol graph has been extended to include action and percepts along with messages.

Messages are passed between agents and percepts/actions are used as the interaction mechanism between agents and actors. We have identified different coverage criteria which include nodes and arcs of the protocol graph. These coverage criteria are used to generate test paths.

For future work, we plan to automate the generation of test data to execute the test paths. Test cases then will be applied to autonomous agents and will uncover the interaction faults. Evaluation to testing technique will show the benefits of applying novel approach in testing of autonomous agents with help of design artifacts following Prometheus methodology.

## Figures and Tables

**Algorithm 1:**
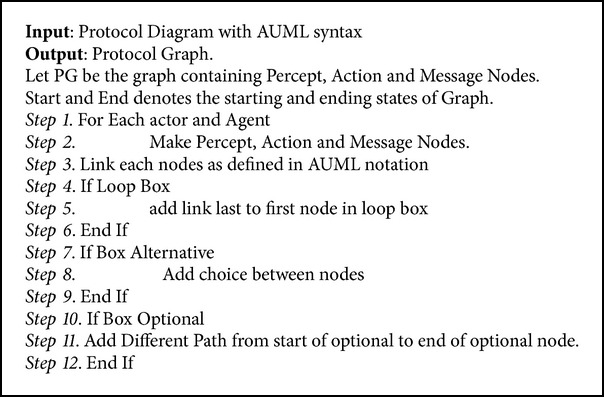
Converting protocol diagram into protocol graph.

**Algorithm 2:**
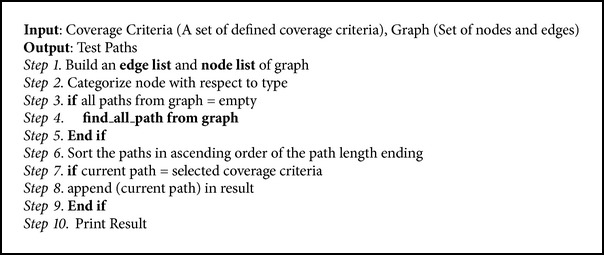
Test path generation from protocol graph.

**Table 1:** Test paths for account operation protocol diagram.

S. #	Coverage criteria	Test paths
1	Message coverage	(i) 1 → 2 → 3 → 4 → 6 (message) → 7 (message) → 8 → 9 (message) → 11 → 13 → 14

2	Action coverage	(i) 1 → 2 → 3 (action) → 5 → 6 → 7 → 8 → 9 → 10 (action) → 13 (action) → 14 (ii) 1 → 2 → 3 → 5 → 6 → 7 → 8 → 9 → 12 (action) → 14 (iii) 1 → 2 → 3 (action) → 4 → 6 → 7 → 8 → 9 → 11 (action) → 13 (action) → 14

3	Percept coverage	(i) 1 → 2 (percept) → 3 → 5 (percept) → 6 → 7 → 8 (percept) → 9 → 10 → 13 → 14 (ii) 1 → 2 (percept) → 3 → 4 (percept) → 6 → 7 → 8 (percept) → 9 → 11 → 13 → 14

4	Message action coverage	(i) 1 → 2 → 3 → 5 → 6 → 7 → 8 → 9 (message) → 10 (action) → 13 → 14 (ii) 1 → 2 → 3 → 5 → 6 → 7 → 8 → 9 (message) → 12 (action) → 14 (iii) 1 → 2 → 3 → 4 → 6 → 7 → 8 → 9 (message) → 11 (action) → 13 → 14

5	Action percept coverage	(i) 1 → 2 → 3 (action) → 5 (percept) → 6 → 7 → 8 → 9 → 10 → 13 → 14 (ii) 1 → 2 → 3 (action) → 4 (percept) → 6 → 7 → 8 → 9 → 11 → 13 → 14 (iii) 1 → 2 → 3 → 5 → 6 → 7 → 8 → 9 → 12 (action) → 5 (Percept) → 6 → 7 → 8 → 9 → 12 → 14 (iv) 1 → 2 → 3 (action) → 4 (percept) → 6 → 7 → 8 → 9 → 11 → 13 → 4 → 6 → 7 → 8 → 9 → 11 → 13 → 14

6	Percept-message coverage	(i) 1 → 2 → 3 → 5 (percept) → 6 (message) → 7 → 8 (percept) → 9 (message) → 10 (action) → 13 → 14 (ii) 1 → 2 → 3 → 4 (percept) → 6 (message) → 7 → 8 (percept) → 9 (message) → 11 (action) → 13 → 14

7	Pairwise-message coverage	(i) 1 → 2 → 3 → 5 → 6 (message) → 7 (message) → 8 → 9 → 10 → 13 → 14

8	All round trip paths	(i) 1 → 2 → 3 → 2 → 3 → 5 → 6 → 7 → 8 → 9 → 10 → 13 → 5 → 12 → 14 (ii) 1 → 2 → 3 → 5 → 11 → 13 → 5 → 12 → 5 → 10 → 13 → 14

9	All paths coverage	(i) Infinite # of Paths

**Table 2:** Test path generator tool input file.

14 start, start account open, precept account created, action debit account request, precept credit account request, precept transport request, message exchange request, message exchange rates, precept exchange request reply, message amount credited, action amount debited, action request error, action accont info, action end, end 27	1,2 2,3 3,2 3,4 3,5 4,6 4,10 4,11 4,12 5,6 5,10 5,11 5,12 6,7 7,8 8,9 9,10 9,11 9,12 10,13 11,13	12,14 12,4 12,5 13,14 13,4 13,5
